# Assessing Therapeutic Choices and Adherence to Antidiabetic Therapy in Naïve Patients: A Retrospective Observational Study in a Local Health Authority of the Piedmont Region (Italy)

**DOI:** 10.3390/healthcare11111655

**Published:** 2023-06-05

**Authors:** Lucrezia Greta Armando, Gianluca Miglio, Raffaella Baroetto Parisi, Mariangela Esiliato, Cristina Rolando, Valeria Vinciguerra, Abdoulaye Diarassouba, Clara Cena

**Affiliations:** 1 Department of Drug Science and Technology, University of Turin, Via Pietro Giuria 11, 10125 Turin, Italy; gianluca.miglio@unito.it (G.M.); clara.cena@unito.it (C.C.); 2 Competence Center of Scientific Computing C3S, University of Turin, Corso Svizzera 185, 10149 Turin, Italy; 3 Struttura Complessa Farmacia Territoriale ASL TO4, Regione Piemonte, Via Nino Costa 43, 10034 Chivasso, Italy; rbaroettoparisi@aslto4.piemonte.it (R.B.P.); mesiliato@aslto4.piemonte.it (M.E.); crolando@aslto4.piemonte.it (C.R.); vvinciguerra@aslto4.piemonte.it (V.V.); adiarassouba@aslto4.piemonte.it (A.D.)

**Keywords:** first-choice antidiabetic therapy, prescription patterns, medication adherence, antidiabetic drugs

## Abstract

Due to its prevalence and socio-economic burden on health systems, diabetes mellitus (DM) is considered a major health emergency. This retrospective, observational study aimed to describe a population of DM-naïve patients of the Local Health Authority (LHA) ASL TO4 Regione Piemonte and the prescriptive behavior of LHA general practitioners. Drug dispensing data collected between January 2018 and December 2021 was analyzed. Adult patients were included if they received their first prescription for an antidiabetic drug (AD) in 2019 and had ≥2 prescriptions/year of ADs during the follow-up. Patients who started antidiabetic therapy with metformin were selected to investigate comorbidities, medication adherence, and first treatment intensification. Comorbidities were identified through a modified version of the Rx-Risk Index; adherence was measured as the continuous measure of medication availability (CMA). Among 1927 DM-naïve patients, 1361 started therapy with metformin. Most of them received drugs related to cardiovascular diseases, hypertension, and infectious diseases during the study period. Median CMA was 58.8%, with the majority of patients being partially adherent to ADs (40 ≤ CMA < 80). Initial antidiabetic therapy was mostly modified (switch, add-on) with SGLT-2 inhibitors and sulfonylureas. These findings help to identify areas of intervention to improve the use of ADs in the LHA.

## 1. Introduction

Diabetes mellitus (DM) is a chronic disease widely spread throughout the world, with more than 537 million people suffering from it. In particular, it is estimated that 9.8% of adults and 1.2 million children and adolescents (less than 0.05% of the world population under 19 years) worldwide suffer from type-2 DM (T2DM) and type-1 DM (T1DM). These numbers are expected to increase to 643 million people with DM by 2030; this growth is mainly due to the progressive aging of the population, and secondarily to the spread of sedentariness, obesity, and socio-economic inequalities [1,2].

The International Diabetes Federation (IDF) calculated a global healthcare expenditure for both T1DM and T2DM of 966 billion dollars, of which a large part is due to the treatment of comorbidities and hospitalizations. As a matter of fact, the relevance of this pathology can be found in the numerous complications that patients may encounter, including diabetic retinopathy, the leading cause of blindness among adults, and diabetic nephropathy, the leading cause of chronic renal failure. Moreover, DM is responsible for about 60% of leg amputations, and it represents the fourth cause of death in the European Union, with an almost double mortality rate in diabetic individuals compared with non-diabetic ones [2]. For this reason, DM is one of the three health emergencies identified by the United Nations (UN) and the World Health Organization (WHO) along with malaria and tuberculosis [2,3,4]. The social and economic burden of DM on national health systems goes beyond the condition itself as the comorbidities that afflict T1DM and T2DM patients strongly impact the ability of patients and healthcare professionals to effectively manage the disease. DM-associated comorbidities can appear before or after the onset of the disease, and they were classified by Piette et al. [5] into concordant and discordant conditions. The former, such as ischemic heart disease and hypertension, are considered concordant to DM because they share the same pathophysiologic risk profile and management goals, while those that are considered discordant disease conditions, such as rheumatologic diseases and depression, are not directly related to the pathogenesis of DM. The latter, despite having a minor impact on treatment options, could influence patient self-care behavior and lifestyle and affect medication adherence [5,6,7].

At the national level, the Italian Istituto nazionale di statistica (Istat) estimated a prevalence of T1DM and T2DM of 5.9%, which increases with aging up to 21% in people over 75 years old; this makes Italy one of the European countries with the highest percentage of diabetic patients [8,9].

Individuals with T1DM are dependent on the administration of exogenous insulin to survive, whereas it is well-established that T2DM can be managed through an optimized antidiabetic therapy, which is essential to reduce elevated blood glucose levels and prevent short and long-term complications. In order to counteract symptoms of hypoglycemia and improve the quality of life of diabetic patients, national and international guidelines for the treatment of T2DM define metformin as the first-choice drug in all age groups regardless of body weight. Other drug classes used as second- or third-line therapies alone or in combination with metformin are the sodium-glucose cotransporter-2 inhibitors (SGLT-2i), the glucagon-like peptide 1 receptor agonists (GLP-1RAs), pioglitazone, the dipeptidyl peptidase-4 inhibitors (DPP-4i), and, in severe cases, insulin. SGLT-2i and GLP-1RAs are also recommended for patients with previous cardiovascular (CV) events, while DPP-4i are particularly suitable for the treatment of elderly patients due to their ease of use and lack of side effects [8,9]. Although still widely prescribed, new evidence has associated sulfonylureas (SUs) and repaglinide with an increased risk of mortality; therefore, their use is discouraged by the most recent guidelines [10].

A valid strategy to improve prescriptive appropriateness and sustainability of care is represented by the analysis of drug dispensing/prescription data, consisting of administrative databases regularly collected by healthcare facilities, which offer a wealth of information at a low cost. As numerous studies have already shown [11,12,13,14,15,16,17,18], the analysis of drug prescriptions at the local or national level can support the identification of prescriptive patterns and patient behavior models useful for effective healthcare planning. In fact, despite the preventive measures implemented in Italy, inadequate antidiabetic drug (ADs) usage is common in terms of both medication adherence and persistence, as well as hospitalizations resulting from non-optimal management of antidiabetic therapy [8]. The Italian Medicines Agency (Agenzia Italiana del Farmaco, AIFA) estimated a 29% rate of subjects with suboptimal adherence to ADs, with a higher percentage of subjects with poor adherence in the over 85 age group. Poor adherence to treatment occurs when patients autonomously stop taking their medications, omit one or more doses, or vary the time of intake from that prescribed. This behavior compromises the safety and effectiveness of antidiabetic treatment and may lead to poor glycemic control, resulting in an increased risk of hospitalizations, increased morbidity, and premature mortality. In addition, poor adherence to ADs has a significant impact on the expenditures of the Italian National Health Service (NHS), since diabetic complications account for about 60% of the total direct costs of the disease (estimated to be 15 billion euros per year) [3,19].

Despite several studies that have analyzed antidiabetic prescription patterns in different patient populations, only a limited number of studies have focused on naïve patients to investigate how drug therapy is implemented [2]. The aim of this retrospective, observational study was to describe how general practitioners (GPs) of the Local Health Authority (LHA) ASL TO4 Regione Piemonte set initial therapy with ADs in a treatment-naïve patient population. Secondary objectives were: (a) to describe changes in antidiabetic therapy (i.e., combination and switching) in a sample of the study patients; and (b) to assess medication adherence to ADs and to investigate factors related to poor therapy management. The study involved patients of the LHA ASL TO4 Regione Piemonte, located in the northern area of the city of Turin (Piedmont Region, Italy), which includes both urban and rural areas and represents 12% of the region’s population.

## 2. Materials and Methods

### 2.1. Data Source

In Italy, essential medications are provided free of charge to all the inhabitants by the LHA where citizens have their regular address. In addition, drug dispensing data are collected monthly for administrative purposes by the same authority. These include patient-level information on drugs reimbursed by the Italian NHS prescribed by GPs and pediatricians.

This study was based on the analysis of drug dispensing data collected between 1 January 2018 and 31 December 2021 by the ASL TO4 Regione Piemonte. In particular, the database contains the records of drugs dispensed by community pharmacies in the corresponding geographical area. The information included in these records consists of the patient’s anonymized code, birth date, and gender, along with the dispensation date, medication name, active ingredient, Anatomical Therapeutic Chemical (ATC) code, number of dispensed packages, and number of Defined Daily Dose (DDD) [20]. Data sources were linked to the LHA registry of deaths in order to collect any date of death.

The study was conducted in accordance with the principles of the General Data Protection Regulation (EU) 2016/679, ensuring full compliance with current privacy legislation. Specifically, the data underwent anonymization from the original source and the authors had no access to personally identifiable patient information. On 21 November 2022, the Ethics Committee of the ASL TO4 Regione Piemonte approved the study.

### 2.2. Study Population

Eligible subjects consisted of individuals aged 18 and older on 31 December 2021 receiving their first prescription for an AD in the calendar year 2019 (index period). The year 2018 was used as a wash-out period, meaning that all individuals who already had at least one dispensation for an AD before 1 January 2019 were excluded; in this way, it was possible to include only patients naïve to antidiabetic treatment. In addition, as ADs are intended for the treatment of a chronic condition, only patients who had at least 1 prescription of an AD in the index period and at least 2 prescriptions of an AD per year during the follow-up were included. The index date corresponded to the dispensation date of the first antidiabetic drug during the specified index period. Patient follow-up extended from the index date until either their last prescription date or the end of the study period. Patients who died by the end of the study period were excluded from the analysis.

The study drugs were identified based on their ATC code, specifically focusing on drugs used in DM (ATC code A10). In Italy, all approved ADs are covered by the Italian NHS, allowing their utilization to be investigated through the analysis of administrative databases.

A modified version of the Rx-Risk Comorbidity Index [21] was used to identify patients’ comorbidities during the study period. The Rx-Risk Index is a validated comorbidity index that only requires prescription/dispensing data using ATC classification codes: when an individual has at least one prescription/dispensation of a specific drug, he or she is considered to be on treatment for that comorbidity [21,22]. The index was adapted for our study to use the ATC codes at level 2 (ATCL2) to map the diseases.

A subgroup of the study population was considered for the assessment of medication adherence: they were patients who started antidiabetic therapy with metformin. This subgroup was studied according to the number of ADs prescribed during their follow-ups. Metformin initiators who received a second AD within 9 days from the index date were categorized as first-line combination therapy initiators.

**Figure 1:**
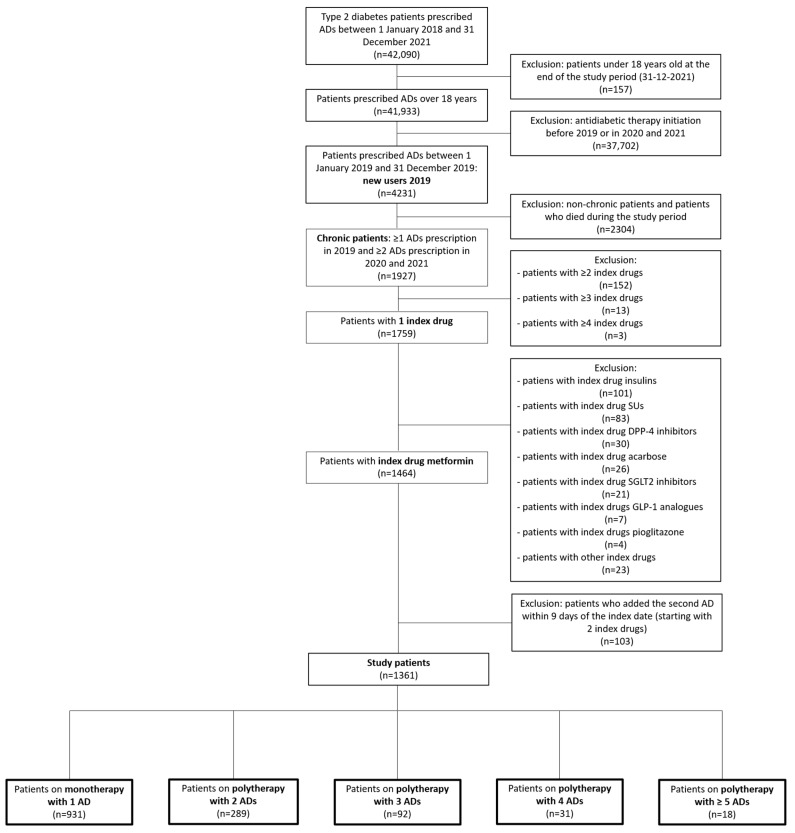
Flow chart of the process of identifying and selecting the study cohort. Abbreviations: ADs, antidiabetic drugs; SUs: sulfonylureas; DPP-4, dipeptidyl peptidase-4; SGLT2, sodium/glucose cotransporter 2; GLP-1, glucagon-like peptide 1.

Progression over time of metformin monotherapy initiators with regard to their AD regimen was monitored in the patient subgroup with 2 different ADs. The first change in AD therapy was considered for the analysis of switch and add-on. A switch was defined as a change in prescription with a drug other than metformin during the follow-up; add-on was defined as either the prescription of a second AD along with metformin during the follow-up or the prescription of a fixed-dose AD combination containing metformin. Figure 1 shows the selection criteria adopted.

### 2.3. Analysis

Baseline demographics, patient characteristics, and treatment-related events (i.e., switch) were reported descriptively. Medication adherence was measured as the continuous multiple-interval measure of medication availability (CMA) during the follow-up. To measure medication adherence, the duration of each dispensation was calculated by dividing the total amount of active substance in each dispensed package by the DDD. The R statistical and programming language (version 4.0.5; https://cran.r-project.org/; accessed on 1 April 2022) was used for the analysis. The following add-on packages and their dependencies were used: tidyverse, eeptools, dplyr, rdrugtrajectory, doBy, ggplot2, AdhereR, survival, survimer, lubridate.

## 3. Results

### 3.1. Baseline Characteristics and First-Line Antidiabetic Therapy

A total of 41,933 individuals aged 18 years and older received an AD during the study period and were potentially eligible. Among them, 1927 (56.6% men) met the inclusion criteria and were enrolled in the study. General characteristics of the study population are summarized in Table 1.

**Figure 2:**
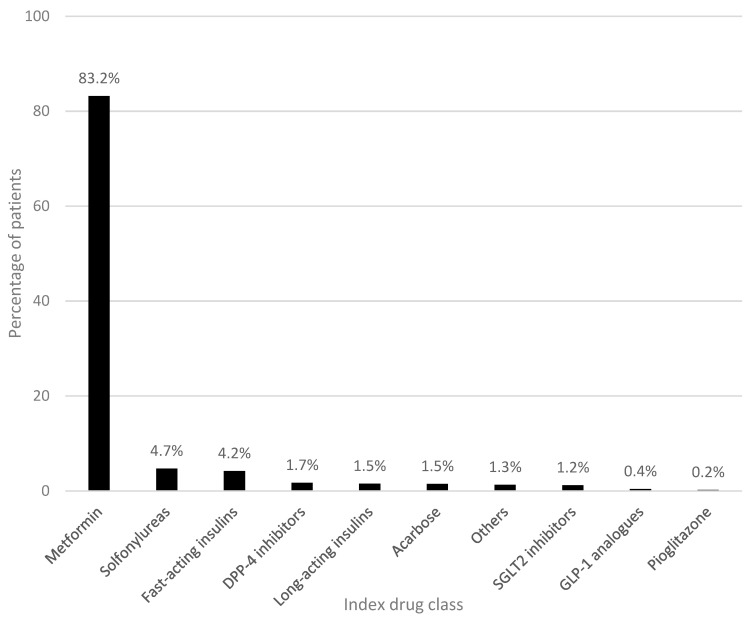
Distribution of patients according to their index drug. Data represent the percentage (frequency) of naïve patients prescribed with 1 AD (*N* = 1759). Abbreviations: DPP-4, dipeptidyl peptidase-4; SGLT2, sodium/glucose cotransporter 2; GLP-1, glucagon-like peptide 1.

The largest subset (91.3%) were patients with one index drug. Metformin was the index drug for the majority of them (83.2%), while GLP-1 analogues and pioglitazone were prescribed as the index drug to less than 1.0% of patients (Figure 2).

In order to better understand how antidiabetic therapy was implemented, the subgroup of patients who started antidiabetic therapy with metformin was divided into 5 subsets according to the number of ADs prescribed during the follow-up. After removing 103 patients who had been prescribed a second AD other than metformin within 9 days of the index date, 1361 patients were considered. Their general characteristics are summarized in Table 2. The largest subset (68.4%) was on metformin monotherapy along the entire follow-up period; at the opposite, less than 2.0% of the sample were prescribed with ≥5 ADs in their study period. The male:female ratio was comparable among the 5 subgroups, while age was significantly (*p* < 0.01) reduced with increasing complexity of antidiabetic therapy.

### 3.2. Identification of Comorbidities Based on Dispensed Drugs

**Figure 3:**
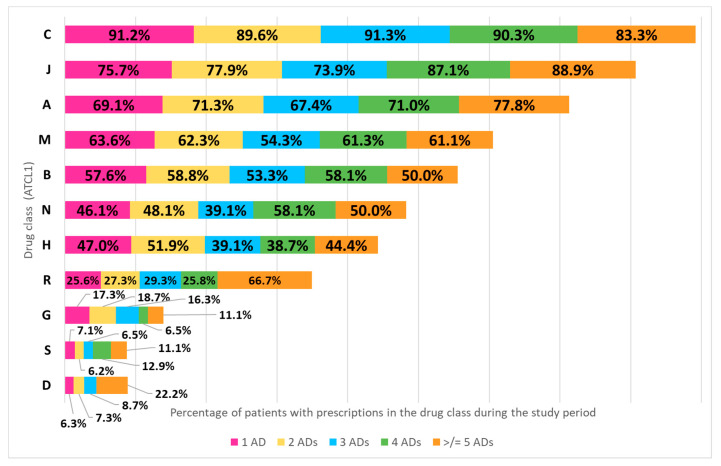
Percentage (frequency) of patients (in each subgroup) with dispensations of drugs other than ADs during the study period according to their ATCL1. Only drug groups at ATCL1 with ≥10% prevalence in at least one subgroup were considered. Abbreviations: AD, antidiabetic drug; C, cardiovascular system; J, antiinfectives for systemic use; A, alimentary tract and metabolism; B, blood and blood forming organs; N, nervous system; H, systemic hormonal preparations; R, respiratory system; G, genito-urinary system and sex hormones; S, sensory organs; D, dermatologicals.

First, all drugs dispensed to the analyzed population were classified according to the 14 ATC groups at level 1 (ATCL1), excluding ATC A10–ADs, in order to investigate which drug classes were dispensed to diabetes-naïve patients who started treatment with metformin (*N* = 1361) during the study period. Figure 3 shows the percentage of patients in each subgroup with prescriptions of drugs of other classes used during the study period. It should be remembered that only medications considered essential by the Italian NHS are recorded in the drug dispensing data analyzed and that no limits were imposed on the number of (co)prescriptions dispensed to the same individual during the study period.

Secondly, comorbidities were identified from the classes of drugs (ATCL2) dispensed to patients during the study period. Comorbidities were then classified as DM-concordant or DM-discordant according to the definition provided by Piette et al. [5]. Table 3 shows the medical conditions included in the adapted version of the Rx-Risk Index with ≥10% prevalence in at least one of the subgroups.

### 3.3. First Treatment Intensification: Add-On and Switch

**Figure 4:**
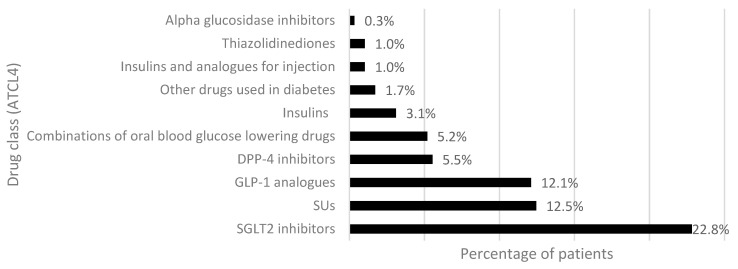
Frequency of drug classes added to metformin. Abbreviations: SGLT2, sodium/glucose cotransporter 2; SUs, sulfonylureas; GLP-1, glucagon-like peptide 1; DPP-4, dipeptidyl peptidase-4.

**Figure 5:**
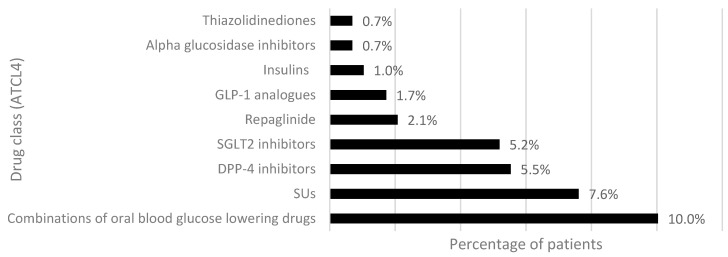
Frequency of drug classes switched from metformin. Abbreviations: SUs, sulfonylureas; DPP-4, dipeptidyl peptidase-4; SGLT2, sodium/glucose cotransporter 2; GLP-1, glucagon-like peptide 1.

The subgroup of patients with 2 ADs was selected to analyze the first intensification of antidiabetic therapy. Out of 289 patients with 2 ADs, 189 (65.4%) added a second drug to metformin, 92 (31.8%) switched to another antidiabetic drug, and 8 (2.8%) discontinued therapy after switching. Figure 4 and Figure 5 show the frequency of drugs added to metformin monotherapy or switched, respectively. The drugs most frequently prescribed together with metformin belonged to the class of SGLT-2i, which is appropriate as a second-line therapy, particularly in subjects with previous CV events. SUs were the second most added drugs to metformin monotherapy, followed by GLP-1RAs and DPP-4i. The drug class at ATC level 4 (ATCL4) most switched from metformin were the fixed-dose combinations of metformin with other oral hypoglycemic agents (metformin/sitagliptin, metformin/dapagliflozin, metformin/empagliflozin, metformin/vildagliptin, metformin/SUs), followed by SUs, DPP-4i, and SGLT-2i. A total of 15 patients, 6.3% of group 1 (add-on) and 3.3% of group 2 (switch), respectively, intensified their therapy with insulins alone or in combination. Subjects who discontinued antidiabetic treatment after switching (*N* = 8) had replaced metformin with SGLT-2i (*N* = 5, 1.7%), SUs (*N* = 2, 0.7%) and GLP-1RAs (*N* = 1, 0.3%).

### 3.4. Adherence to Antidiabetic Therapy

**Figure 6:**
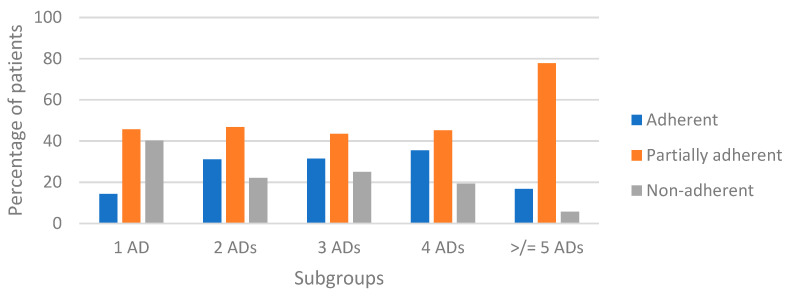
Stratification of study patients based on adherence. Abbreviation: AD, antidiabetic drug.

To better understand how ADs were used in our study population, medication adherence was evaluated. Patients were stratified according to their CMA value into adherent (CMA ≥ 80%), partially adherent (40% ≤ CMA < 80%), and non-adherent (CMA < 40%) [18]. The percentage of patients partially adherent to antidiabetic medication was the highest subset in all subgroups. As shown in Figure 6, as the complexity of antidiabetic therapy increases, the percentage of adherent patients increases and that of non-adherent patients decreases, with the exception of the last subgroup (≥5 ADs). Table 4 summarizes the results of the medication adherence analysis. Median medication adherence was lower for the monotherapy subgroup (46.0%), while it was comparable for the other subgroups (mean medication adherence excluding the monotherapy subgroup 62.0%).

## 4. Discussion

DM represents a major public health issue due to its prevalence, especially among the elderly, the associated complications, and related direct and indirect costs [16,23,24].

This study explored some aspects of AD utilization in a population of treatment-naïve patients in an area in the province of Turin (Piedmont Region, Italy). Despite the availability of national and international guidelines on the use of ADs in adults and elderly, discrepancies exist between guidelines and actual clinical practice [16,23,24]. The analysis of drug dispensing data provides real-world evidence on the utilization of ADs in the ASL TO4 Regione Piemonte and could be helpful to better understand physician prescription behavior and patient preferences. Our results may lead to the identification of unmet needs and areas of intervention for healthcare systems.

As recommended by national guidelines [9], metformin was the drug most commonly prescribed as the initial antidiabetic treatment (83.2%). In second place were the SUs (4.7%), which the latest evidence advises against in preference of other drug classes such as SGLT-2i and GLP-1RAs [10]. In contrast to other European studies [24,25], only a small percentage of patients (1.3%) started antidiabetic therapy with repaglinide. Moreover, unlike previous studies [26], we observed a limited number of first-line prescriptions of the more recently introduced ADs: DPP-4i (1.7%), SGLT-2i (1.2%), and GLP-1RAs (0.4%). This can be explained by the way these drug classes were prescribed in the Piedmont Region; before 2022, in fact, GPs could not prescribe them, so patients had to be seen by a diabetologist to get them. Of note, the use of SGLT-2i and GLP-1RAs was associated with a significant decrease in CV deaths, and therefore they were more recently recommended as an alternative to metformin in patients with previous CV events [10]. On the other hand, patients who started antidiabetic therapy with fast-acting insulins (4.2%) and long-acting insulins (1.5%) can be traced back to T1DM.

In our study cohort (patients with metformin as index drug, *N* = 1361), males were the majority in all 5 subgroups, reflecting national data on the prevalence of DM in Italy [3,4]. The median age of the study population was 69.0 years [IQR 60.0–76.0], with a significant difference depending on the total number of ADs prescribed during the study period. In fact, the median age decreases with the increasing complexity of antidiabetic therapy, with younger patients (median age 62.0 years [IQR 53.0–72.0]) belonging to the ≥5 AD subgroup. This could indicate that older patients generally follow established therapies, while younger ones have more frequent adjustments of the antidiabetic therapy.

The results of the present study suggest that metformin monotherapy was the main treatment option (68.4% of the study cohort) adopted in the ASL TO4 Regione Piemonte, while fixed-dose combination therapies and the use of ≥2 ADs was reserved to a lower percentage of patients (31.6%); this could be explained by the limited follow-up period, as patients could remain stable on first-line therapy for several years before the progression of the disease. Collectively, these findings indicate that the prescription of ADs in our study population could be influenced by factors related to the patient’s age and clinical condition.

Based on the drugs dispensed, all patients in the 5 subgroups had concomitant conditions associated with the use of drugs acting on the cardiovascular system (ATC C01, C04–C08) and of antithrombotic agents (B01), while antihypertensives (C02, C03, C09) were prescribed to the majority of patients during the study period. These results are consistent with other studies carried out in Europe [7,22,27], where authors found high proportions of diabetic patients with cardiovascular/cerebrovascular disease and hypertension. The other DM-concordant conditions (hyperlipidemia–C10– and hyperuricemia/gout–M04) also presented similar percentages in the studies conducted by O’Shea et al. [22] and by Guerrero-Fernández de Alba et al. [27]. With regard to DM-discordant conditions, the main differences with previous studies [7,22,27] concerned the use of antiinfectives (J01–J07) and of anti-inflammatory products (M01–M03, M09): we found that almost the majority of patients in each subgroup used antiinfectives and anti-inflammatory drugs during the study period. This difference could be due to the fact that previous studies only considered chronic concomitant diseases and not acute conditions generally treated with drugs prescribed for limited periods. The number of patients with a given comorbidity was comparable in the 5 subgroups, with the exception of chronic obstructive airways diseases (R03), gastrointestinal disorders/nausea (A03, A04, A06, A07), epilepsy (N03), and dermatological diseases (D01–D11), for which a higher prevalence was observed in the ≥5 AD subgroup. This may indicate that patients who adjusted their antidiabetic therapy more frequently during the study period (≥5 ADs) are a subgroup of patients typically younger and with more prescriptions for drugs of different classes by their GP, which may be associated with a higher number of acute or chronic comorbidities.

The analysis of therapy intensification by switching or adding other ADs revealed that patients starting antidiabetic therapy with metformin often added or replaced metformin with SUs. SUs reduce hemoglobin A1c (HbA1c) similar to metformin, but they increase body weight and are associated with an increased risk of hypoglycemia [23]. Notably, the prescription of newer ADs as a second-line therapy was still limited in the ASL TO4 Regione Piemonte in 2019, but is expected to increase in the coming months thanks to the introduction of the “Nota AIFA 100” [28] in January 2022, which allows GPs to prescribe SGLT-2i, GLP-1RAs, and DPP-4i to all diabetic patients at no additional cost to the citizen, as well as the publishing of the most recent national guidelines at the end of 2021 [10].

Overall rates of adherence in people of the ASL TO4 Regione Piemonte with DM are suboptimal, especially in the subgroup of monotherapy patients. With increasing complexity of antidiabetic therapy, the percentage of adherent patients (CMA ≥ 80%) increases and that of non-adherent patients (CMA < 40%) decreases, with the exception of the subgroup with ≥5 ADs. In general, more complex therapies could harm patients’ adherent behavior. These results are confirmed by the scientific literature [19,29]. For instance, a recent study [26] conducted in Italy showed an average adherence of 67%, which the authors associated with a higher probability of hospitalizations and mortality.

### Strengths and Limitations

The main strength of the present study is that it provides robust real-world evidence from a large administrative database showing the patterns of use of ADs in the population of the ASL TO4 Regione Piemonte.

As with other research on the use of drugs, some limitations of the study should be noted. Firstly, drug dispensing data are not collected for research purposes and, consequently, some misclassification can occur. Secondly, CMA is a useful proxy for adherence but it may not always accurately reflect actual medication-taking behavior. Finally, the unavailability of clinical information does not allow the correlation of poor adherence and persistence to clinical outcomes that would allow for further stratification of the population.

## 5. Conclusions

To conclude, the choice of antidiabetic therapy in the ASL TO4 Regione Piemonte is variable and can be influenced by the age of the patients and the presence of comorbidities. Even if, in general, the guidelines are followed, a number of patients are being treated with SUs, which have been associated with an increased risk of mortality and should, therefore, be chosen with greater caution.

The findings of the present study allow the identification of unmet needs and areas of intervention aimed at improving the use of ADs in the ASL TO4 Regione Piemonte, but their validity can certainly be extended outside the study area to provide an overview on the real use of ADs.

## Figures and Tables

**Table 1:** General characteristics of the study population.

Study population, *N*	1927
Males, *N* (%)	1090 (56.6)
Age, median [IQR]	67.0 [58.0–75.0]
Males	66.0 [57.0–73.0]
Females	70.0 [60.0–78.0]
Number of index drugs, *N* (%)	
1 index drug	1759 (91.3)
2 index drugs	152 (7.9)
3 index drugs	13 (0.7)
≥4 index drugs	3 (0.2)

Abbreviation: IQR, interquartile range.

**Table 2:** General characteristics of the study cohort.

Characteristics *N* = 1361	1 AD	2 ADs	3 ADs	4 ADs	≥5 ADs	*p*-Value
Patients, *N* (%)	931 (68.4)	289 (21.1)	92 (6.8)	31 (2.3)	18 (1.3)	
Gender, *N* (%)						0.14
Males	488 (52.4)	176 (60.9)	54 (58.7)	18 (58.1)	10 (55.6)	
Age, median [IQR]	71.0 [62.0–78.0]	65.0 [57.0–74.0]	64.5 [54.2–73.0]	64.5 [54.2–73.0]	62.0 [53.0–72.0]	<0.01
Males	69.0 [62.0–75.0]	65.0 [56.0–73.0]	62.0 [53.0–70.0]	62.0 [53.0–70.0]	58.0 [53.0–73.0]	
Females	72.0 [62.0–80.0]	67.0 [60.0–75.2]	66.0 [60.0–74.0]	66.0 [60.0–74.0]	64.0 [59.0–69.0]	
Drugs of other classes used during the study period, median [IQR]	10.0 [6.0–15.0]	11.0 [7.0–17.0]	9.0 [6.0–15.0]	12.0 [6.5–18.5]	14.0 [7.7–17.5]	0.13
Males	9.0 [5.0–15.0]	10.0 [5.7–15.0]	9.0 [5.0–10.0]	9.5 [7.0–17.0]	15.0 [13.0–22.5]	
Females	12.0 [8.0–17.0]	13.0 [8.0–19.0]	9.5 [7.0–16.7]	16.0 [6.0–22.0]	9.0 [6.0–16.0]	

Chi-square test and the one-way ANOVA test were used, when appropriate. Abbreviations: AD, antidiabetic drug; IQR, interquartile range.

**Table 3:** Percentage of patients (frequency) in each subgroup with comorbidities according to the adapted version of the Rx-Risk Index. Only comorbidities with ≥10% prevalence in at least one subgroup were considered.

Drug Classes (ATCL2)	Related Medical Conditions	Patients with Prescriptions in the Drug Class during the Study Period (%)				
		1 AD	2 ADs	3 ADs	4 ADs	≥5 ADs
DM-concordant conditions						
C01, C04, C05, C07, C08, B01	Cardiovascular/cerebrovascular diseases and heart diseases	100.0	100.0	100.0	100.0	100.0
C02, C03, C09	Hypertension	100.0	100.0	94.6	100.0	77.8
C10	Hyperlipidemia	62.5	69.6	80.4	64.5	61.1
M04	Hyperuricemia/Gout	18.0	20.4	13.0	16.1	11.1
DM-discordant conditions						
J01–J07	Infectious diseases	83.8	87.5	88.0	100.0	100.0
A02	Acid related disorders	57.1	60.2	54.3	67.7	77.8
M01–M03, M09	Inflammatory/Rheumatic disorders	56.6	52.6	43.5	48.4	55.6
H02	Corticosteroid-responsive diseases	40.7	45.7	33.7	32.3	44.4
A08–A09, A11–A16	Nutrition-related diseases	36.5	32.9	29.3	29.0	33.3
N02	Pain, including migraine	29.9	29.4	23.9	41.9	33.3
N06, N07	Depression and other mental disorders	21.4	21.1	20.7	19.4	27.8
R03	Chronic obstructive airways diseases	19.9	20.8	23.9	22.6	61.1
A03–A04, A06–A07	Gastrointestinal disorders and nausea	19.9	19.4	18.5	22.6	50.0
B03	Anemia	17.7	17.3	9.8	6.5	11.1
G01–G04	Diseases of the genito-urinary system, including benign prostatic hypertrophy	17.4	19.0	16.3	6.5	11.1
H03	Thyroid disorders	12.5	13.5	7.6	9.7	11.1
R01–R02, R05–R07	Respiratory diseases	10.2	9.7	10.9	3.2	11.1
N03	Epilepsy	9.7	13.5	5.4	29.0	27.8
D01–D11	Dermatological diseases, including psoriasis	7.3	8.3	9.8	0.0	22.2
S01, S03	Eye disorders, including glaucoma	7.1	6.2	6.5	12.9	11.1
N05	Psychotic illnesses	3.3	5.5	3.3	6.5	11.1

Abbreviations: AD, antidiabetic drug; C01, cardiac therapy; C04, peripheral vasodilators; C05, vasoprotectives; C07, beta blocking agents; C08, calcium channel blockers; B01, antithrombotic agents; C02, antihypertensives; C03, diuretics; C09, agents acting on the renin-angiotensin system; C10, lipid-modifying agents; M04, antigout preparations; J01, antibacterials for systemic use; J07, vaccines; A02, drugs for acid-related disorders; M01, anti-inflammatory and antirheumatic products; M03, muscle relaxants; M09, other drugs for disorders of the muscolo-skeletal system; H02, corticosteroid for systemic use; A08, anti-obesity preparations; A09, digestives, including enzymes; A11, vitamins; A16, other alimentary tract and metabolism products; N02, analgesics; N06, psychoanaleptics; N07, other nervous system drugs; A03, drugs for functional gastrointestinal disorders; A04, antiemetics and antinauseants; A06, drugs for constipation; A07, antidiarrheals, intestinal anti-inflammatory/anti-infective agents; R03, drugs for obstructive airway diseases; B03, antianemic preparations; G01, gynecological antiinfectives and antiseptics; G04, urologicals; H03, thyroid therapy; R01, nasal preparations; R02, throat preparations; R05, cough and cold preparations; R07, other respiratory system products; N03, antiepileptics; D01, antifungals for dermatological use; D11, other dermatological preparations; S01, ophthalmologicals; S03, ophthalmological and otological preparations; N05, psycholeptics.

**Table 4:** Adherence to ADs.

	1 AD	2 ADs	3 ADs	4 ADs	≥5 ADs
	Median [IQR]	Median [IQR]	Median [IQR]	Median [IQR]	Median [IQR]
Adherence to ADs	46.0 [31.0–64.0]	60.0 [41.0–84.0]	62.0 [41.0–82.0]	63.0 [52.0–83.0]	63.0 [49.0–75.0]
Males	47.0 [33.0–66.0]	60.0 [41.0–84.0]	64.0 [42.0–82.0]	61.0 [47.0–81.0]	61.0 [49.0–71.0]
Females	43.0 [29.0–61.0]	60.0 [44.0–84.0]	58.0 [36.0–76.0]	66.0 [54.0–85.0]	64.0 [51.0–79.0]

Abbreviations: AD, antidiabetic drug; IQR, interquartile range.

## Data Availability

Not applicable.
